# Design and Effect of Resonant Ultrasonic Vibration-Assisted Laser Cladding (R-UVALC) on AlCrFeMnNi High-Entropy Alloy

**DOI:** 10.3390/ma17050969

**Published:** 2024-02-20

**Authors:** Aziz Ul Hassan Mohsan, Mina Zhang, Dafeng Wang, Yishen Wang, Jiahao Zhang, Yanyuan Zhou, Yifei Li, Su Zhao

**Affiliations:** 1 Ningbo Institute of Materials Technology and Engineering, Chinese Academy of Sciences, Ningbo 315201, China; hassan@nimte.ac.cn (A.U.H.M.); wangyishen@nimte.ac.cn (Y.W.); zhangjiahao@nimte.ac.cn (J.Z.); zhouzyy123@hrbeu.edu.cn (Y.Z.); liyifei@nimte.ac.cn (Y.L.); 2 International College, University of Chinese Academy of Sciences, Beijing 101408, China; 3 Ningbo Branch of Chinese Academy of Ordnance Science, Ningbo 315103, China; bjing2013saw@126.com

**Keywords:** resonant ultrasonic vibration assisted laser cladding (R-UVALC), high-entropy alloy, FEA, micrograph and microstructure, mechanical and tribology properties

## Abstract

The design of the resonant ultrasonic vibration-assisted laser cladding (R-UVALC) setup involved employing finite element analysis (FEA) to simulate the ultrasonic transducer, horn, and workpiece in a resonance state. The impact of R-UVALC on AlCrFeMnNi high-entropy alloys was assessed using various ultrasonic vibration amplitudes of 0, 5, 10, and 15 µm, with a constant frequency of 20 kHz. Ultrasonic vibrations reduced pores and cracks and increased the clad breadth, melt pool wetting angle, and laser-clad layer consistency. The columnar elongated grains in proximity to the substrate surface underwent a size reduction and transformed into grains with a more equiaxed shape with the utilization of ultrasonic vibrations at an amplitude of 5 µm. Laser cladding performed without ultrasonic vibrations yields two phases: face-centered cubic (FCC) and body-centered cubic (BCC). However, when the coating is exposed to ultrasonic vibrations with an amplitude of 5 µm, it forms a solitary body-centered cubic (BCC) phase. The microhardness tripled compared to the substrate, and the most significant microhardness value was achieved at 5 µm of ultrasonic vibration. The friction coefficient was assessed at an ambient temperature, revealing that an ultrasonic amplitude yields the lowest friction coefficient, demonstrating the excellent wear resistance properties of the coating. The analysis of the 3D surface profile of the wear indicates that the use of ultrasonic aid with a 5 µm amplitude leads to reduced depth of scars, and the primary wear mechanism observed is abrasive and oxidative wear with fewer grooves and debris. In addition, XPS analysis revealed the presence of metal components in an oxidized condition, suggesting that the wear process is oxidative in nature. Integrating the R-UVALC setup into a resonance state can significantly enhance the efficiency of the laser cladding process in the laser cladding field.

## 1. Introduction

Laser cladding (LC) is a method employed to improve the wear resistance and restoration of engineered alloys by reinforcing and restoring the surface. Nevertheless, a significant concern regarding laser cladding coatings is the non-uniform dispersion of reinforcements and the occurrence of fractures, both of which have a substantial impact on the qualities of the coating. Novel techniques employing optimized laser processing parameters and powdered alloy components have been devised to tackle this issue [1,2,3,4]. Preheating and post-heating are techniques employed to produce crack-free coatings in laser cladding. However, the utilization of these techniques is limited. Alternative methodologies need to be explored, and ultrasonic vibration is a particularly promising technique to address the limitations of the laser cladding process [5,6,7,8,9,10,11,12].

Multiple studies have been undertaken using ultrasonic vibration-assisted laser cladding. Zhou et al. [13] performed studies on Inconel 718; the micrographs and mechanical properties of material produced using ultrasonic vibration-assisted laser cladding were evaluated using amplitudes of 10, 15, 20, and 25 µm. Li et al. [14] performed UVALC tests on Ni/WC/La_2_O_3_, with ultrasonic power of 600, 700, and 900 W. An evaluation was conducted on the microstructure and properties, revealing that UVALC contributes to the improvement in both the microstructure and mechanical parameters. Another study examined the mechanical properties of TiC/TiB coatings at different ultrasonic power levels (200, 400, and 600 W). The findings showed that the microstructure and mechanical and tribological properties were enhanced when subjected to ultrasonic vibration-assisted laser cladding (UVALC) [15]. D-D Zhuang et al. [16] investigated the effect of ultrasonic vibrations and their action process on the microstructure and mechanical performance of 316L Steel under UVALC. Amplitudes of 15, 17.5, and 20 µm were applied. The ideal choice was identified as improved mechanical performance with an amplitude of 17.5 µm. All of these studies have concentrated on investigating the impact of ultrasonic vibrations on the UVALC. However, they have neglected to adequately design the ultrasonic system [6,16,17,18,19]. Additionally, these studies have employed ultrasonic vibrations without considering the integration of the workpiece as part of the ultrasonic system. Consequently, the unequal ultrasonic vibration generated in the melt pool makes it challenging to control and replicate the output results. Excluding the workpiece from the ultrasonic system greatly reduces the possibility of achieving optimal effectiveness of the ultrasonic vibrations.

The setup for resonant ultrasonic vibration-assisted laser cladding (R-UVALC) includes laser cladding equipment that has been improved with the addition of an ultrasonic transducer, a horn, and a regulating mechanism. The controlling mechanism comprises an ultrasonic generator and a laser vibrometer. The ultrasonic generator produces a signal to the ultrasonic transducer to achieve a specific frequency and amplitude. The laser vibrometer is utilized to ascertain the constant frequency and amplitude of the ultrasonic transducer. The ultrasonic vibrations produced by the piezoelectric transducer are insufficient to achieve the needed amplitude. To overcome this limitation, an ultrasonic horn amplifies the amplitude by bringing the ultrasonic horn into a resonant state. Ultrasonic horns were previously developed analytically and refined by building and testing experimental prototypes [20]. Analytical models simplify horn representation by solving simple wave equations to determine geometric dimensions. Both analytical and experimental prototyping techniques possess inherent limits, particularly when dealing with intricate designs. Engaging in experimental prototyping requires a significant amount of time. Considerable effort was required to make minor design alterations throughout the development and production stages. FEA analysis is utilized due to its flexibility, speed, cost-effectiveness, and ability to handle intricate geometries [21,22,23,24].

The ultrasonic horn and workpiece are designed to operate at a resonance frequency for maximum vibration amplitude. The system is advanced using the first longitudinal vibration mode. The system’s initial longitudinal mode has the maximum output amplitude. Patel et al. [25] employed ANSYS software to conduct modal and simple harmonic analysis on a hybrid horn. The hybrid yielded the highest amplification factor while also reducing the corresponding stresses. Amin et al. [26] employed a CAD model and FEM analysis to design the ultrasonic horn for the objective of ultrasonic machining of difficult-to-machine materials. Multiple studies have been undertaken on the design of ultrasonic horns for diverse purposes, particularly in ultrasonic machining and ultrasonic plastic welding [20,27,28,29]. Currently, there is no existing research that can be utilized to develop an ultrasonic setup specifically for ultrasonic vibration-assisted laser cladding in a resonance state.

High-entropy alloys (HEAs) are formed by combining uniform or significant amounts of (often) five or extra elements, commonly resulting in simple solid solutions (FCC, HCP, and BCC) rather than intricate intermetallic compounds. HEA exhibits impressive combinations of high strength, extraordinary resistance to thermal softening, outstanding wear resistance, and corrosion resistance [30,31,32]. Presently, a multitude of High-entropy Alloy (HEA) coatings are employed in the process of laser cladding onto 304 steel. The coatings mentioned include AlCoCrFeNiNbx [33], FeCoNiCrMnTi [34] Al_x_CoCrFeNi [35], Fe_50_Mn_30_Co_10_Cr_10_ [36], FeCrFeNiNb_x_ [37], FeNiCoCrMo_x_ [38], and AlCoCrFeNi [39]. The application of laser-clad HEA coatings has demonstrated substantial scientific significance in enhancing the surface characteristics of stainless-steel substrates. The study of equiatomic FeCoCrMnNi alloys has received considerable scholarly attention within the materials discipline exploration community [40,41,42]. The alloys have superior resistance to chemical corrosion and possess exceptional ductility. The alloys mostly consist of a single-phase face-centered cubic (FCC) solid solution. The nature of the alloys limits their capability in mechanical applications that require both eminent strength and exceptional wear resistance properties. The exorbitant price of Co metal also restricts the affordability of the alloys and hampers their widespread implementation in the industry. In order to achieve high-entropy alloys (HEAs) with well-balanced mechanical properties, several investigators have introduced aluminum (Al) into the single-phase face-centered cubic (FCC) alloys. The inclusion of Al in the alloy matrix facilitates the development of a robust body-centered cubic (BCC) phase and improves the mechanical durability with good wear resistance properties of the alloys [43,44].

The laser cladding process for AlCrFeMnNi is complex due to its inherent brittleness and susceptibility to cracking. The laser cladding of AlCrFeMnNi will likely result in various phases, specifically FCC and BCC. Ultrasonic Vibration-Assisted Laser Cladding (UVALC) induces thermal, acoustic streaming, and cavitation effects, resulting in fluctuations in the undercooling temperature and nucleation activity throughout the solidification process [45]. As a result, the grains are improved, and the dissolution of crystalline grains leads to improved microstructural and mechanical properties. Inadequate investigation has been conducted on the UVALC design and its impact on the micrograph, microstructure, and mechanical properties of the AlCrFeMnNi HEA alloy [19,46]. This study aims to examine how different levels of ultrasonic vibrations affect the micrographs, microstructure, friction coefficient, and microhardness of the material while keeping the entire system in a resonant condition of one direction.

## 2. Methodology

### 2.1. Design of Ultrasonic Horn and Workpiece

**Figure 1:**
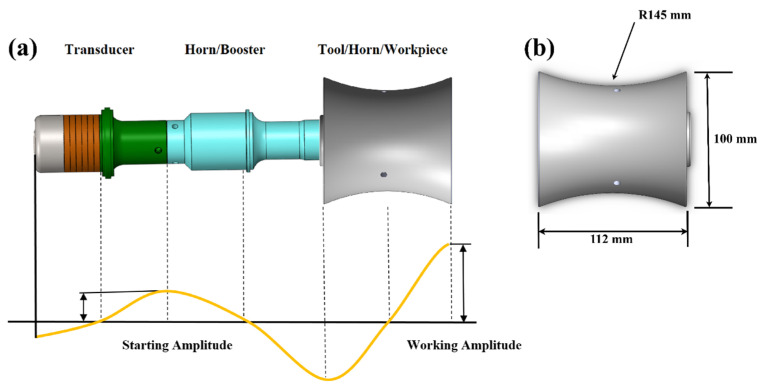
(**a**) Schematic of half wavelength ultrasonic transducer, ultrasonic horn, and workpiece design for Resonant Ultrasonic Vibration-Assissted Laser Cladding (R-UVALC), (**b**) Schematic of substrate/workpiece design and dimensions for R-UVALC.

Prior studies have investigated ultrasonic horns using analytical and finite element modal analysis [47,48]. The stepped horn provides the highest degree of magnification. The length was approximated using plane wave equations and subsequently adjusted to fall within the range of 115 mm to 130 mm, in accordance with the intended amplification factor form and resonant frequency requirements. The laser cladding setup utilized a substrate made of 304 steel, which has a Young’s modulus (Y) of 190 GPa and a density (⍴) of 8000 kg/m^3^. The approximate length of the substrate was determined using wave equations. The ultrasonic horn and workpiece were modified in accordance with the ultrasonic transducer. The half-wavelength ultrasonic design and workpiece/substrate dimensions are shown in Figure 1a,b, respectively. The entire system was created using Solidworks 2021 and then imported into Ansys for modal analysis.

### 2.2. Finite Element Analysis (FEA)

The transducer horn and workpiece analysis is performed using the modal and harmonic response analysis features of ANSYS 19.0 software. Equation (1) describes the motion equation that characterizes the free vibration of an acoustic horn when modeled using a finite element (FEA) approach.

(1) $$M\ddot{U}+BÅ®+KU=0$$

In this context, the symbols B, M, and K represent the damping, mass, and stiffness matrix, respectively. Additionally, $\ddot{U}$, Å®, and *U* describe the acceleration, velocity, and displacement of the vibrating body, respectively. By disregarding the effects of air damping, the equation governing the motion can be reformulated as Equation (2),

(2) $$M\ddot{U}+KU=0$$

The modal characteristics of a horn can be assessed by solving the Eigenvalue problem in the following manner shown in Equation (3).

(3) $$\left(K-\omega_{i}^{2}M\right)\varphi_{i}=0$$

$\varphi_{i}$ is the i^th^ eigenvector according to the mode shape, while $\omega_{i}$ is the natural frequency of i^th^ mode shape. The method has the potential to encompass several natural frequencies and their related mode shapes within a specified frequency scale.

#### Modal and Harmonic Response Analysis

The modal analysis function of ANSYS Workbench 19 is utilized to conduct modal extraction for the horn, and frequency response analysis was conducted. In the initial stage, the material geometry and material properties were specified. Subsequently, the global coordinate system was chosen. In the third step, the mesh was generated to facilitate FEA analysis. The mesh generation process employed a modal analysis mesh feature, which automatically adjusted the element size to optimize computational efficiency. Every individual constituent possesses distinct characteristics such as stress stiffening, plasticity, and the ability to undergo enormous strains.

**Figure 2:**
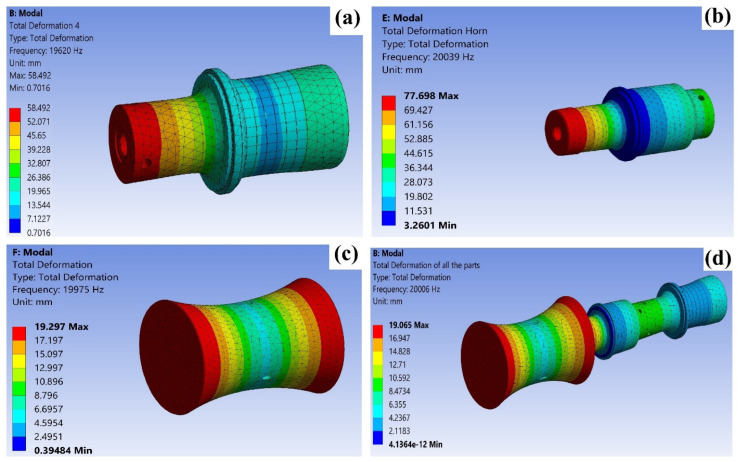
Frequency response analysis of ultrasonic horn with total deformation at 20 kHz frequency: (**a**) ultrasonic transducer, (**b**) ultrasonic horn, (**c**) workpiece, and (**d**) all of the parts.

The mesh creation modes were retrieved using the Block Lanchoz approach. In the context of modal analysis, an initial selection of 50 modes was made in order to visually represent the range of modal alternatives available at various frequencies. Additionally, a restriction was imposed on the frequency range between 19 and 21 kHz in order to isolate the desired modes at the specified frequency. The longitudinal mode was selected at a precise frequency of 20 kHz. Figure 2a–d depicts the whole ultrasonic system analysis consisting of the ultrasonic transducer, horn, and workpiece.

The ultrasonic horn and workpiece were manufactured and thereafter connected to the self-assembled ultrasonic transducer operating at a frequency of 20 kHz. The Impedance Analyzer SinoSonics PV520 (1 kHz~1 MHz) was utilized to measure the impedance, and impedance matching was performed in line with the specified specifications. In addition, the Laser Doppler Vibrometer (LDV) SOPTOP LV-S01 was utilized to measure the amplitude and frequency. The obtained results fell within the predetermined amplitude range of 0–30 µm and a frequency of 20 kHz with an approximation error of 5%.

## 3. Experimental Methods

### 3.1. Formulation of the HEA Coatings

**Figure 3:**
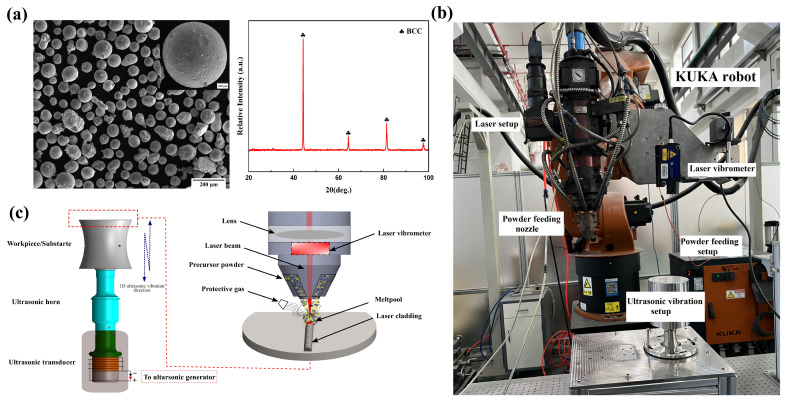
(**a**) SEM micrograph and XRD pattern of AlCrFeMnNi high-entropy alloy powder, (**b**) schematic of the experimental setup, (**c**) and actual experimental setup of resonant ultrasonic vibration-assisted laser cladding.

The high-entropy alloy (HEA) powders used in this study were produced using gas atomization. Figure 3a illustrates that the pre-alloyed powders exhibit a predominantly sphere shape, with a size range spanning from 45 to 145 μm. The powders predominantly comprise a single body-centered cubic (BCC) solid solution phase, as seen by the X-ray diffraction (XRD) patterns shown in Figure 3a. Table 1 displays the nominal chemical makeup of the powders, confirming that the powders include five primary alloying components in identical molar ratios. The process of laser cladding coatings was performed on the surface of the 304 stainless-steel substrates by following the specifications determined by the modal analysis. The workpiece, as shown in Figure 1b, had a diameter of 100 mm and a length of 112 mm. A compound system consisting of a laser device, a programmable automated arm (KUKA), a cladding head, and a self-developed ultrasonic setup was used to produce ultrasonic vibration-assisted laser cladding of HEA coatings. The KUKA reboot system makes use of a coaxial powder feeding nozzle, and its highest power outturn is 4000 W. The ultrasonic-assisted setup employs a consistent frequency of 20 kHz to transmit ultrasonic vibrations. The studies were conducted both without ultrasonic vibration (WOU) and with ultrasonic vibration (WU) amplitudes of 5, 10, and 15 µm. To improve the precision of vibration amplitude during the R-UVALC, a laser vibrometer was linked to the KUKA robot to measure ultrasonic vibrations at each step. The schematic and real diagram of the experimental arrangement are shown in Figure 3b,c, respectively. A set of first investigative studies was carried out without the inclusion of the ultrasonic effect to refine the processing parameters. The laser was set to a power of 2100 W, with a cladding rate of 7 mm/s and a flow rate of 7 L/min. The laser spot diameter used was 3 mm. Argon gas was used at a flow rate of 15 L/min throughout the experiments to avoid oxidation during the cladding process.

### 3.2. Materials Characterization

The samples underwent mechanical grinding, progressing from rougher sandpaper to finer sandpaper until they achieved a grit size of 5000. This procedure was conducted to examine the microstructure of the materials. This was then followed by diamond polishing to enhance the visualization of the microstructure. The samples were subjected to etching using Aqua regia, precisely with a mixture of hydrochloric acid (HCL), nitric acid (HNO_3_), and water (H_2_O) in a ratio of 3:1:6 for a duration of 30 s. The X-ray diffraction (XRD) examination was performed utilizing CuKα radiation with a wavelength of 1.78897 Å, employing a D8 Advance Davinci apparatus manufactured by Bruker (Billerica, MA, USA). The analysis aimed to ascertain the phase composition of the coated surface. The instrument was utilized with a voltage of 35 kV and a current of 40 mA. The powders and coatings underwent analysis using a scanning electron microscope (SEM, FEI Quanta 250 FEG) that was equipped with energy-dispersive spectroscopy (EDS). The analysis aimed to evaluate the microstructure and chemical composition of the samples. The coatings’ cross-sections were subjected to microhardness testing utilizing a Vickers indenter with a 100 g load and a dwell period of 15 s. The microhardness value was determined by taking the average of three measurements for each sample. Each test was conducted at intervals of 100 µm until the desired substrate values were reached. Friction testing was performed under dry conditions utilizing a GF-I multifunctional material surface performance tester at an ambient temperature. The counterpart consists of round Si_3_N_4_ material with a diameter of 4 mm. The force exerted is 10 N, with a rotational speed of 280 r/min, and it lasted for a duration of 20 min. Furthermore, the debris present on the worn track was analyzed using X-ray photoelectron spectroscopy (XPS) with the AXIS ULTRA DLD instrument manufactured by Kratos (San Diego, CA, USA).

## 4. Results and Discussion

### 4.1. Effect of Ultrasonic Vibrations on the Macro Morphologies

**Figure 4:**
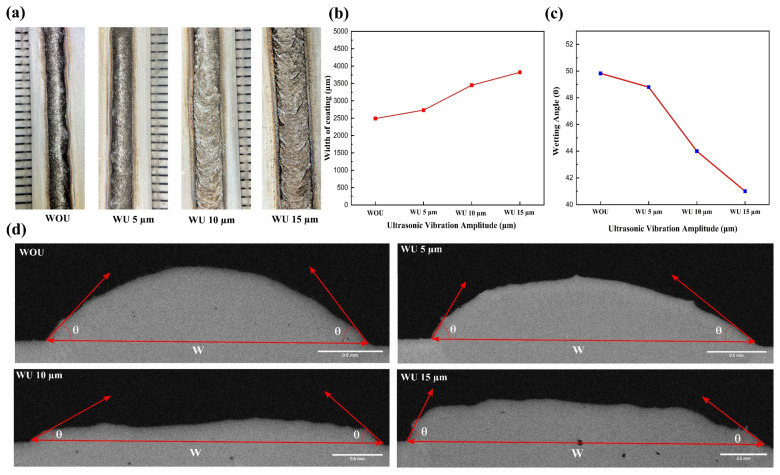
(**a**) Micrographs of surface profile without ultrasonic and with ultrasonic vibration amplitudes of 5, 10, and 15 µm; (**b**) width of the coating WOU and WU amplitudes; (**c**) wetting angle (average values of both sides) of the coating WOU and WU amplitudes; (**d**) analysis of the coating’s cross-sectional structure under different ultrasonic amplitudes, without and with ultrasonic amplitudes of 5, 10, and 15 µm.

Figure 4a displays the laser-clad coating profiles at different ultrasonic amplitudes: without ultrasonic and with ultrasonic vibration amplitudes of 5 µm, 10 µm, and 15 µm, respectively. The coating applied without using ultrasonic vibration shows an uneven distribution along the cladding path. In contrast, the cladding performed under ultrasonic vibration has a smoother profile, reduced waviness, fewer cracks, and an excellent connection to the substrate. During the solidification procedure, the homogeneous microstructure results from the ultrasonic acoustic and cavitation effects on the melting pole. Under the influence of ultrasonic vibration, the powder particles underwent complete melting. In contrast, without the aid of ultrasonic assistance, there was a noticeable absence of fusion and the presence of tiny cracks inside the coating. Laser cladding technology is classified as a non-equilibrium phase transformation process, characterized by rapid melting and quick solidification. The heightened temperature gradient during cladding induced residual stress in the coating, hence amplifying the susceptibility to cracks in HEA coatings. The coating cladded without ultrasonic exhibits the presence of micropores and cracks, which are also visible even at higher ultrasonic amplitude. Figure 4d illustrates the different cross-sectional morphologies of the cladding at different amplitudes. The cross-sectional profiles reveal a decrease in the number of pores when subjected to ultrasonic vibrations at an amplitude of 5 µm, compared to when no ultrasonic vibrations are applied. The rapid movement of liquid material in the molten pool and the appropriate release of gas in the molten pool are facilitated by force created by the volume and the pressure gradient formed by ultrasonic vibrations in the direction of their propagation. The width of the coating is denoted as W in the cladding, and the wetting angle is θ as shown in Figure 4b,c, respectively.

When transitioning from no ultrasonic vibration to an ultrasonic vibration with an amplitude of 5 µm, the wetting angle progressively declines, as shown in Figure 4c. However, interestingly, when the vibration amplitude exceeds 5 µm, a distinct pattern appears. In this pattern, the wetting angle experiences a significant decrease once the amplitude surpasses 5 µm. The metal droplet’s profile changes due to the combined effects of gravity and surface tension, moving in a direction where mechanical energy reduces and internal energy increases. With an amplitude of 5 µm, the vibration energy is insufficient to surpass the elastic deformation of the droplet. As a result, the shape and position of the melt-pole droplet almost remain unchanged. At the 5 µm vibration frequency, the energy of the vibrations was insufficient to surpass the energy of adhesion between the melting pole and the substrate [49]. As the amplitude increases, the energy of the vibrations becomes greater than the energy of adhesion. This causes the molten droplet to not separate but instead expand on the substrate. From Figure 4b,c, it can be observed that the width of the coating increases as the wetting angle decreases, and this expansion can also be observed in the cross-sectional profile of the laser-clad coating at different ultrasonic amplitudes as shown in Figure 4d. Furthermore, increasing the ultrasonic amplitude even more leads to further expansion of the molten droplet. Increasing the amplitude even further causes the high-entropy alloy particles to be dispersed outside the coating area through the laser cladding process.

### 4.2. Phase Analysis

**Figure 5:**
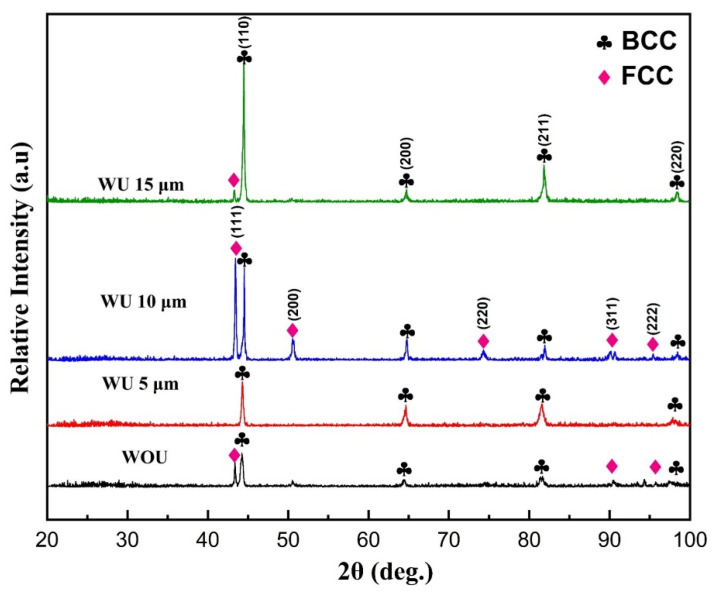
XRD pattern of R-UVALC of HEA AlCrFeMnNi without and with ultrasonic amplitudes of 5 µm, 10 µm, and 15 µm.

Figure 5 displays the X-ray diffraction (XRD) patterns of the AlCrFeMnNi high-entropy alloy (HEA) coatings that were produced using various ultrasonic vibration amplitudes. All coatings display diffraction peaks that may be identified by their body-centered cubic (BCC) and face-centered cubic (FCC) structures, except when the vibration amplitude is 5 µm. In laser cladding without ultrasonic vibration assistance, two distinct phases are observed due to inadequate melting and uneven distribution of high-entropy alloy (HEA) particles in the molten pool during the melting and solidifying process. The FCC and BCC lattice parameters were identified as 2.88 and 3.59, respectively. However, when 5 µm of ultrasonic vibration is applied, a single body-centered cubic (BCC) solid solution structure is achieved, similar to the pre-alloyed powder, as shown in Figure 3a. The presence of the solitary BCC phase at a vibration amplitude of 5 µm is accredited to the combined impact of increased mixing entropy and rapid cooling degree caused by acoustic streaming and cavitation processes. Another reason for the transition from FCC to BCC structure at 5 µm can be attributed to the increase in aluminum (Al) content percentage compared to the without ultrasonic vibrations. This is because Al has a relatively large atomic radius, which leads to lattice distortion and the displacement of some atoms, resulting in the formation of a new BCC lattice structure. Similar observations have been reported in the literature by Wang et al. for the AlxCoCrFeNi high-entropy alloy [50]. Conversely, when ultrasonic vibrations have an amplitude above 5 µm, they cause the evaporation of elements with relatively large atomic radii, such as aluminum (Al) and manganese (Mn), resulting in greater lattice deformation. This can also be observed at the EDS of the profile at 10 and 15 µm in the microstructure portion. Acoustic cavitation enhances the absorption energy of power particles from the laser, resulting in a rise in temperature at the melting pole. As a result, aluminum (Al) and manganese (Mn) evaporate from the pool because they have a lower melting point and vaporization heat associated with the other elements, as shown in Table 2. Additionally, a decrease in the quantity of aluminum content is directed to the existence of the face-centered cubic (FCC) phase, which aligns with the findings of Stepanove et al. [43]. Remarkably, ultrasonic vibrations with an amplitude of 10 µm demonstrate that the prevailing phase structure is FCC, as seen by the prominent peak found at 10 µm. The diffraction peaks at the 15 µm amplitude comprise both BCC and FCC phases.

### 4.3. Microstructure Evolution

**Figure 6:**
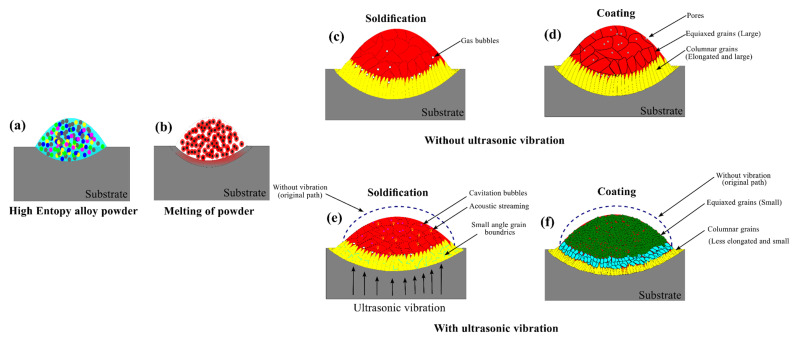
Schematic of grain formation mechanism of laser clad coating with and without ultrasonic vibration, (**a**) high-entropy alloy powder deposition on the substrate, (**b**) melting of high-entropy alloy powder particles, (**c**,**d**) solidification and coating of the profile without ultrasonic vibration, (**e**,**f**) solidification and coating of the profile with ultrasonic vibration.

**Figure 7:**
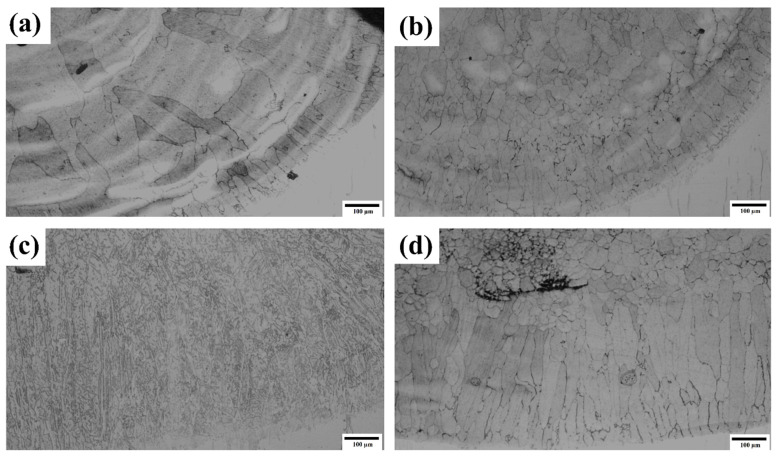
Microstructure of HEA R-UVALC coating near the substrate (**a**) without ultrasonic, (**b**) with ultrasonic of 5 µm, (**c**) with ultrasonic of 10 µm, and (**d**) with ultrasonic of 15 µm.

The process of grain formation under the influence of ultrasonic vibration and without ultrasonic vibration is depicted in Figure 6a–f. The grain growth in the HEA coatings was found to occur from the substrate towards the top of the coatings, mostly because of the relatively low temperature of the substrate, as shown in Figure 6a–d. When influenced by ultrasonic vibrations, the solidification process leads to the formation of smaller grains with an equiaxed grain structure. Additionally, there is a reduced presence of columnar grains at the interface with the substrate. This is because of acoustic streaming and acoustic cavitation. These effects also enhance the formation of small-angle grain boundaries, which in turn promotes nucleation and the generation of small grains, as depicted in Figure 6e,f. Figure 7a depicts the microstructure of the coating without ultrasonic vibration. Notably, the microstructure of the lower sections and certain intermediate parts exhibited columnar elongated grains growing upwards from the bottom. The coating produced without ultrasonic vibration primes to the creation of larger grain sizes on the top and middle sections of the coated surface, as well as larger columnar elongated grains near the substrate. This is attributed to the thermal gradients between the substrate and the coating [51]. The coating exhibited an average grain size within the range of 120–150 µm. Conversely, ultrasonic vibrations lead to the observation of a distinct structure at varying amplitudes. At 5 µm, a smaller grain size throughout the surface of the coating was observed. The coating was accompanied by small equiaxed grains at the top and middle of the coating and smaller-sized columnar elongated grains near the substrate, as shown in Figure 7b. With an amplitude of 5 µm, the grain size was diminished to 40–50 µm, which is roughly half the grain size observed in the absence of ultrasonic vibrations. This phenomenon happened due to the acoustic streaming caused by ultrasonic vibration, which compelled the molten metal to combine and merge, leading to a more uniform distribution of crystal grains and the formation of equiaxed grains. Furthermore, the persistent residual thermal stresses, influenced by high-frequency vibration, potentially facilitated the even distribution of crystals. Another key aspect was the impact of cavitation on microstructures during the process of solidification. The application of ultrasonic vibration in molten metal created many tiny bubble nuclei [13]. At a specific threshold of sound pressure, these bubbles rapidly extended and subsequently collapsed, resulting in the generation of many rapid shockwaves within the molten metal. The shock energy caused the expanding grains to break partially, leading to the scattering of several tiny grains inside the molten pool. Subsequently, there is an increased abundance of new nuclei and smaller grain granules. Moreover, the impact of ultrasound on the rate of nucleation indicates that amplifying the amplitude leads to the swift generation of intense high pressure within the solid solution, thus resulting in a more precise reduction in particle size. This implies that cavitation is a highly effective technique for enhancing the quality of grains during the process of solidification. A distinct crystal structure is observed when the amplitude is increased to 10 µm, as shown in Figure 7c. This structure consists of dendritic elongated cells with branches expanded from the center of the dendrite. An elongated structure was discovered near the bottom of the substrate, whereas the center and top of the surface showed branches that were less elongated. At an amplitude of 15 µm, the crystal structure exhibits a combination of 10 µm and 5 µm structures, with a small number of grains concentrated in the center and elongated precipitation similar to the 10 µm structure. Additionally, smaller equiaxed grains are seen at the top and middle. Observations revealed the presence of larger, elongated columnar grains predominantly located in the lower portion of the clad coating near the substrate, as depicted in Figure 7d.

**Figure 8:**
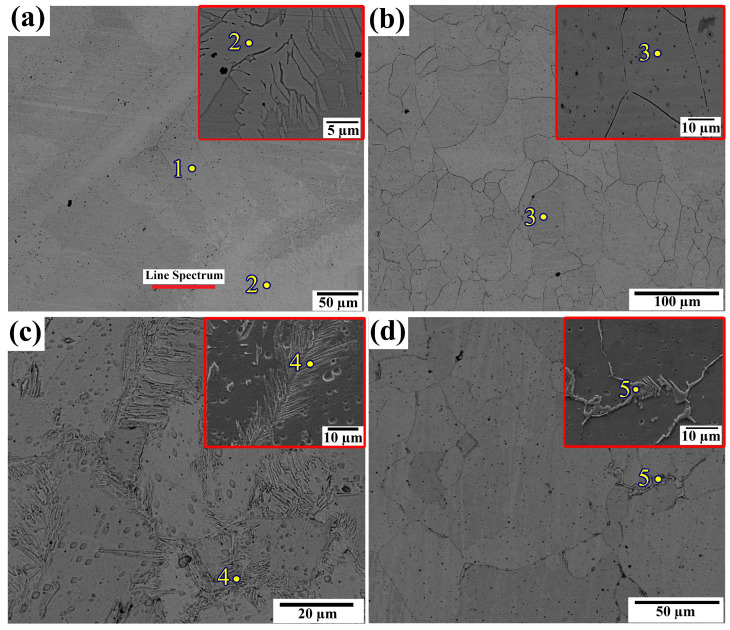
Microstructure of HEA R-UVALC coating: (**a**) microstructure without ultrasonic vibration with point EDS at 1 and point EDS of the closed subset of point 2, (**b**) microstructure with ultrasonic vibration amplitude of 5 µm with point EDS at 3 and closed subset of point 3, (**c**) microstructure with ultrasonic vibration amplitude of 10 µm with point EDS at 4 and closed subset of point 4, and (**d**) microstructure with ultrasonic vibration amplitude of 15 µm with point EDS at 5 and closed subset of point 5; EDS line spectrum marked as line spectrum inside (**a**).

**Figure 9:**
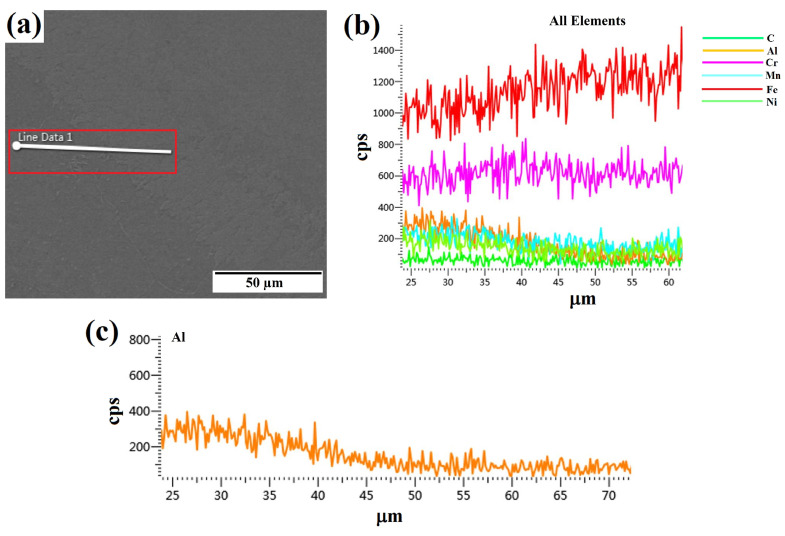
Line spectrum EDS analysis of the portion of two phases present without ultrasonic cladding: (**a**) line EDS on the surface of the R-UVALC coating without ultrasonic vibration, (**b**) elements distribution along the EDS line, and (**c**) Al distribution along the EDS line.

EDS analysis was conducted to visually depict the elemental makeup of the coating’s distinct phases of FCC and BCC. Figure 8a depicts the microstructure of the coating without ultrasonic vibration, showing the presence of two distinct phases in the clad coating. EDS analysis was performed at points 1 and 2 within each phase. Point 1 (BBC phase) was seen to have a larger concentration of Al, whereas point 2 (FCC) had a lower concentration of Al as shown in Table 3. This finding aligns with Stepanov’s [43] finding that the aluminum component plays a crucial part in the conversion between the face-centered cubic (FCC) and body-centered cubic (BCC) phases and vice versa. In order to enhance the understanding of the transition of elements between different phases, a line mapping analysis was performed, as depicted in Figure 9a line spectrum. The findings of the mapping are depicted in Figure 9a–c and unequivocally demonstrate a decrease in the concentration of Al contents when the line transitions between different phases.

The absence of ultrasonic vibration during the laser-cladding process leads to the formation of two distinct phases due to inadequate heat and particle dispersion. A homogeneous single phase was detected throughout the cladding, with an average grain size of 60 µm at an amplitude of 5 µm. An EDS analysis was also performed on the single phase at point 3, which showed that an increased concentration of aluminum (10%) leads to the creation of a BCC phase. The presence of a uniform structure achieved via the use of ultrasonic vibration serves as proof of the efficacy of acoustic streaming and cavitation effects. Continued escalation of ultrasonic vibration at 10 µm leads to the coexistence of two phases. The EDS analysis of the face-centered cubic (FCC) phase matched that of point 2, where ultrasonic effects were absent, indicating a reduced amount of aluminum (Al) element. The FCC occupied a more significant proportion than the BCC at an amplitude of 10 µm. Further increasing the amplitude to 15 µm resulted in two distinct phases with a large portion of BCC and a small portion of FCC. This indicates that the microstructure during the ultrasonic vibration-assisted laser cladding of AlCrFeMnNi high-entropy Alloy (HEA) is greatly affected by the magnitude of ultrasonic vibration.

**Figure 10:**
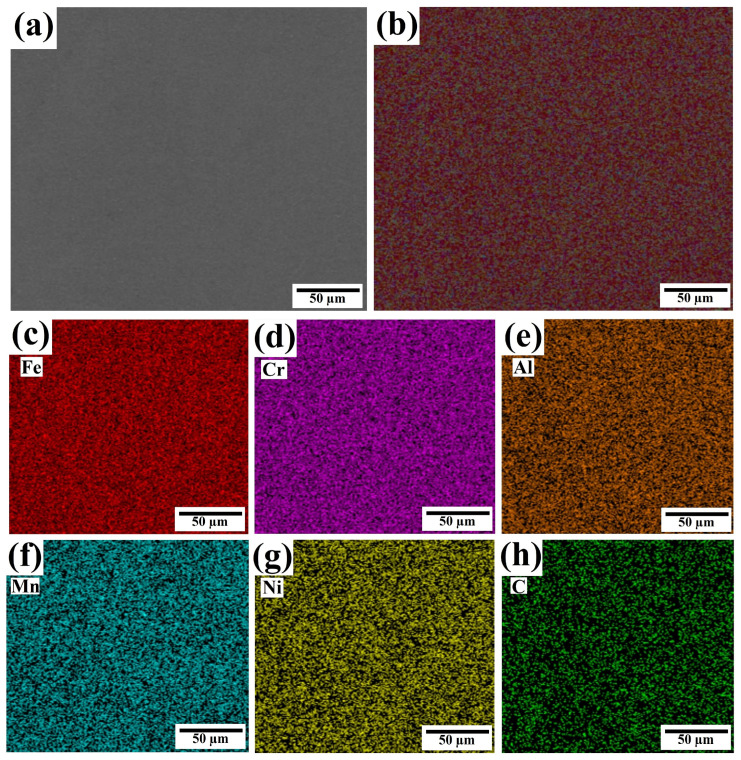
EDS mapping of the R-UVALC coating at 5 µm ultrasonic amplitude, (**a**) selected portion of the R-UVALC coating surface, (**b**) element distribution mapping of the selected portion of the coating, and (**c**–**h**) element distribution mapping of each element Fe, Cr, Al, Mn, Ni, and C, respectively.

EDS mapping was performed on a significant section of the coating cladded at an ultrasonic amplitude of 5 µm, as depicted in Figure 10. An even distribution of all elements is visible with no apparent elemental segregation, indicating that a vibration amplitude of 5 µm is optimal for achieving a homogeneous coating and maximizing the performance of the ultrasonic vibration system.

## 5. Microhardness

**Figure 11:**
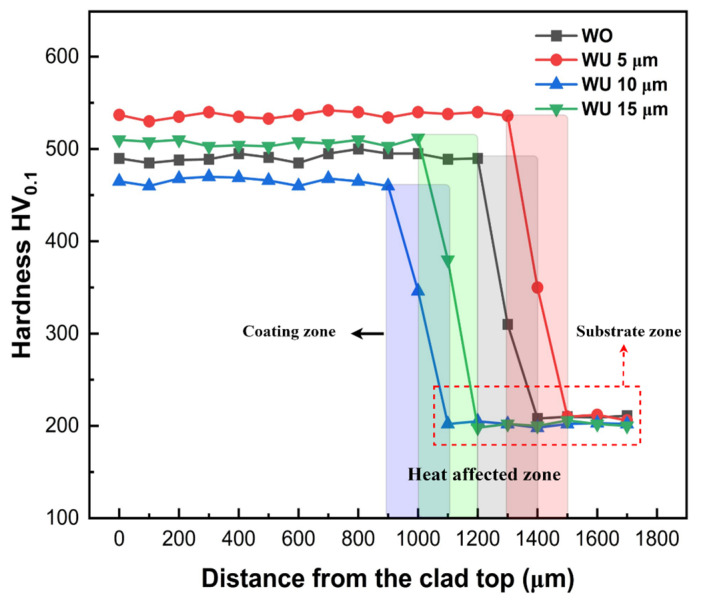
Cross-sectional microhardness profile of HEA R-UVALC coating along the distance from the clad top, without ultrasonic vibration and with ultrasonic vibration amplitudes of 5–15 µm.

Figure 11 depicts the microhardness profile in a cross-sectional view, showcasing different ultrasonic vibration amplitudes. The microhardness of the laser-clad coating has been tripled compared to the microhardness value of the substrate. The microhardness values exhibit the most significant value at the cladding zone, followed by an intermediate level at the heat-affected zone, and the lowest value at the substrate zone. The absence of ultrasonic vibration results in a decreased microhardness compared to the presence of ultrasound at amplitudes of 5 and 15 µm. Microhardness was found to be the lowest at a scale of 10 µm, whereas it was seen to be the maximum at a scale of 5 µm. Acoustic cavitation effects, characterized by a 5 µm amplitude, enhance the fluidity of the molten pool and facilitate a more even distribution of elements inside the cladding layer. The microhardness is maximized when a single solid solution of the BCC phase is present, as observed in the XRD and SEM-EDS analyses. The ultrasonic cavitation effect causes the formation of small grains in the cladding layer, resulting in an enhanced microhardness of the coating compared to when the ultrasonic effect is absent. This effect can be attributed to the Hall–Petch formula, which establishes a direct relationship between the hardness of a material and the decrease in particle diameter [52]. The reduction in porosity at a scale of 5 µm amplitude contributes to the higher microhardness [53]. Conversely, the lowest microhardness was recorded at 10 µm of ultrasonic amplitude. This can be attributed to a higher concentration of the FCC phase, which has lower microhardness values [54]. When subjected to ultrasonic vibration of 15 µm, the presence of a higher proportion of BCC phases and a smaller number of FCC phases results in the second-highest microhardness value.

## 6. Friction Coefficient and Wear Mechanism

**Figure 12:**
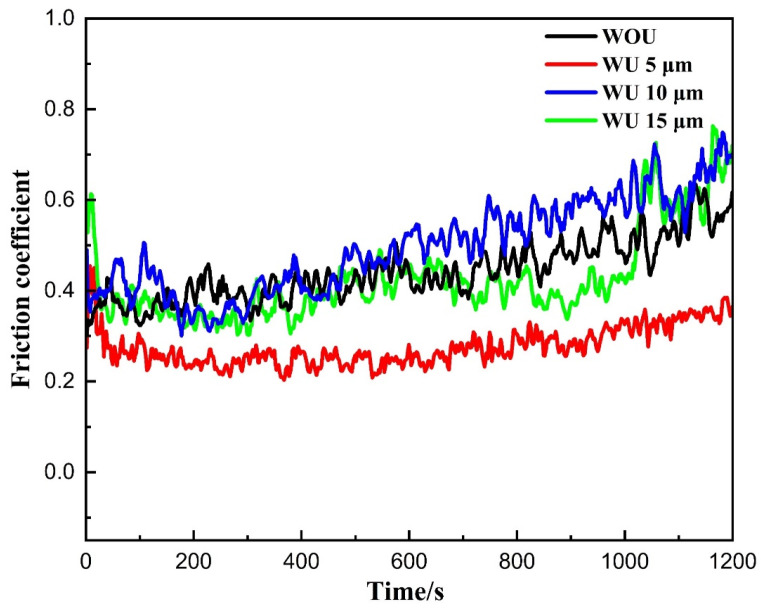
Friction coefficient pattern of HEA R-UVALC coating at ambient temperature with an interval of 1200 s, without ultrasonic vibration and with ultrasonic vibration amplitudes of 5, 10, and 15 µm.

The friction coefficient is an essential parameter used to measure the tribological characteristics of laser cladding layers. The friction coefficient was assessed at an ambient temperature across four distinct scenarios: the absence of ultrasonic vibrations and the presence of ultrasonic vibrations at amplitudes of 5, 10, and 15 µm. The smallest friction coefficient was achieved at an amplitude of 5 µm, but higher ultrasonic amplitudes led to increased friction coefficients. Additionally, a uniform coefficient of friction was measured at an ultrasonic vibration amplitude of 5 µm, as shown in Figure 12. The lowest friction coefficient values are observed at an amplitude of 5 µm, indicating that the coatings demonstrate outstanding antifriction capabilities. The lower friction coefficient of the coatings, produced with an ultrasonic vibration amplitude of 5 µm, can be attributed to the refinement of grains and the formation of a single BCC solid solution phase. This lower friction coefficient is in line with the higher microhardness observed, which reduces the contact area between the rubbing ball and the coating surface, ultimately decreasing the friction coefficient of the coatings. Nevertheless, when the ultrasonic amplitude reached 10 and 15 µm, the coefficient values increased, suggesting an upward trend in the friction coefficient. As previously explained, the increase in friction coefficient at greater amplitudes can be attributed to the creation of both face-centered cubic (FCC) and body-centered cubic (BCC) phases at higher amplitudes. The high coefficient of friction can be strongly correlated with the microhardness profile.

**Figure 13:**
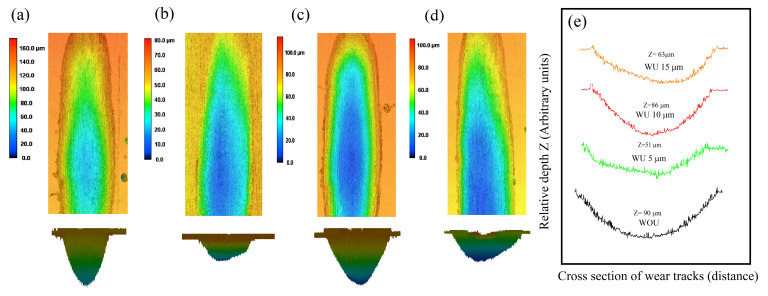
3D surface profile of the wear track produced (**a**) without ultrasonic vibration and with ultrasonic vibration amplitudes of (**b**) 5 µm (**c**) 10 µm, and (**d**) 15 µm, and (**e**) wear scar depth (z) along the cross-sectional wear tracks distance of each of the wear tracks.

In order to obtain a better understanding of the tribological performance, an analysis of the 3D surface morphology of the worn surface was conducted. Figure 13a–d displays the three-dimensional surface characteristics of the worn surface and wear scars of samples subjected to ultrasound at amplitudes of 5, 10, and 15 µm, as well as samples without ultrasound. The wear depth was measured without ultrasound and with ultrasound of various ultrasonic amplitudes of 5, 10, and 15 µm, resulting in depths of 90, 51, 86, and 63 µm, respectively. Both the absence of ultrasonic amplitude and a higher level of ultrasonic amplitude led to more pronounced wear scars, with the minimum wear depth recorded at an amplitude of 5 µm. Ultrasonic vibration significantly affects the wear performance of the coating. A 5 µm amplitude is found to be the most effective in achieving favorable antifriction qualities.

**Figure 14:**
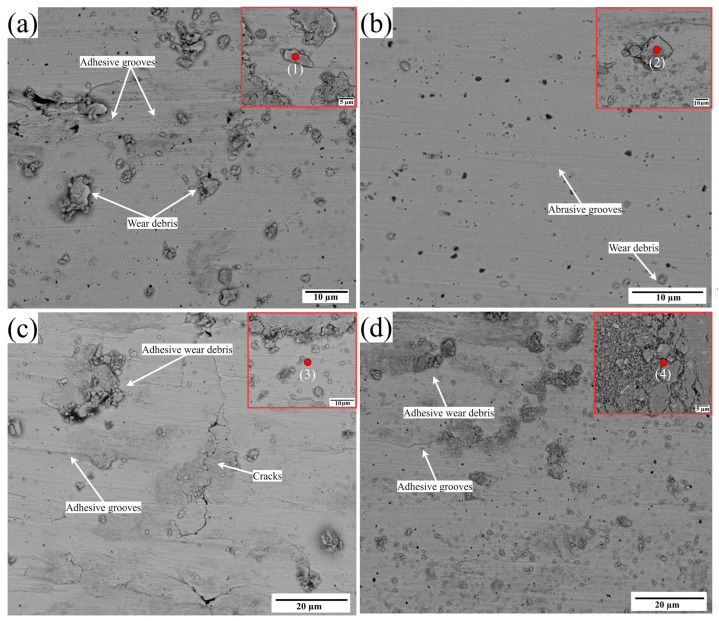
SEM morphology and EDS analysis of wear surfaces and wear debris (**a**) without ultrasonic with point EDS of subset at point 1 and with ultrasonic vibration amplitudes of (**b**) 5 µm with point EDS of subset at point 2, (**c**) 10 µm with point EDS of subset at point 3, and (**d**) 15 µm with point EDS of subset at point 4.

To gain a deeper comprehension of the wear mechanism, the worn surfaces and debris are examined using scanning electron microscopy (SEM) and energy-dispersive X-ray spectroscopy (EDS). The analysis is depicted in Figure 14a–d and Table 4, correspondingly. Figure 14a illustrates that in the absence of ultrasonic vibration, the cladding exhibits greater peeling and more prominent grooves, accompanied by more significant quantities of debris. The subset of Figure 14a point 1 EDS analysis reveals a reduced presence of oxides, indicating the occurrence of adhesive wear. At an amplitude of 5 µm, plastic deformation is reduced and the abrasive action causes tearing and grooving, with smaller debris and grooves. EDS of point 2 shows that there is a higher level of oxidation, indicating that the dominant wear mechanism is abrasive wear. An additional increase in ultrasonic amplitude of 10 µm leads to the formation of more adhesive grooves and the accumulation of more adhesive debris. EDS of point 3 indicates a reduced degree of oxidative wear. A further increase in the ultrasonic amplitude to 15 µm leads to a reduction in the quantity of adhesive debris. EDS analysis of point 4 reveals a moderate oxidation level, indicating that the wear mechanism at 15 µm involves adhesive and abrasive mechanisms.

**Figure 15:**
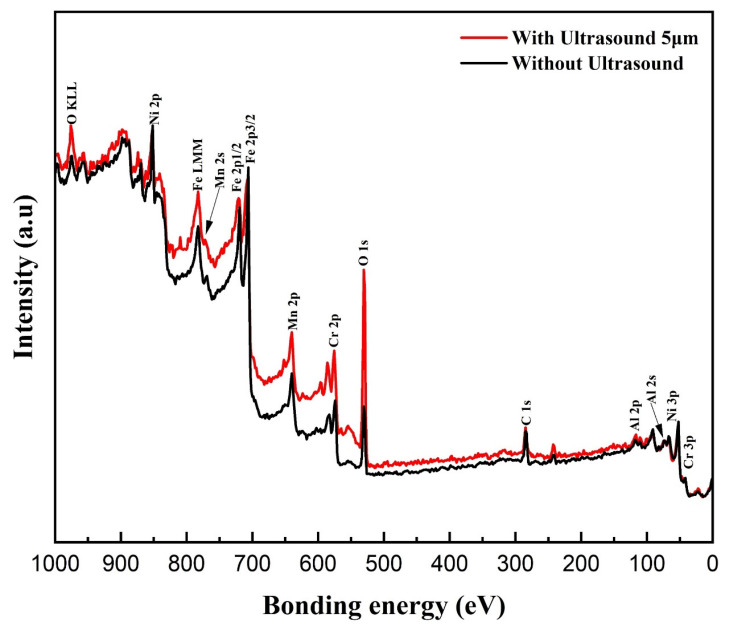
XPS spectra showing the composition and valence state of worn track of the friction testing samples without ultrasonic vibration and with ultrasonic vibration amplitude of 5 µm.

The presence of an oxide film on the surface that experiences wear significantly affects the performance of the coatings in terms of wear. An XPS study was performed to examine the composition and valence state of the oxide coating. The findings are presented in Figure 15. An ultrasonic amplitude of 5 μm leads to a significantly stronger O peak compared to the Al, Cr, Fe, Ni, and Mn peaks. This indicates the existence of metallic oxides comprising these metal elements on the worn surface, which further reveals that the wear process at 5 µm is abrasive and oxidative due to the presence of a robust oxide coating on the worn surface.

## 7. Conclusions

1. FEA ANSYS Modal and frequency response analysis helped in the design of the resonant ultrasonic vibration-assisted laser cladding setup. The system was specifically engineered to resonate with each component, with each component playing a role in creating this condition. At an R-UVALC of 5 µm, there is a claimed enhancement in uniformity, reduced waviness, decreased porosity, fewer cracks, and the presence of a single-phase BCC (body-centered cubic) structure. The lack of ultrasonic cladding leads to the formation of micropores and cracks in the coating that contains two distinct phases. These imperfections persist even at higher ultrasonic amplitudes. At 5 µm R-UVALC single-phase solid solution of BCC was sustained due to the increased mixing entropy and rapid cooling rate, brought about by ultrasonic streaming and cavitation processes.
2. Microstructure WOU results in larger grain sizes at the upper and middle of the coating with elongated columnar grains at the lower part of the coating with an average grain size of 150–160 µm. Conversely, at a 5 µm amplitude, grain size is reduced at the top and middle sections, and smaller columnar elongated grains at the bottom with an average grain size of 60 µm. At 10 µm, the columnar dendrite pattern was observed with an elongated pattern at the bottom and middle of the coating, and a higher portion of FCC and lower of BCC was observed. At 15 µm, a smaller equiaxed grain size was observed in the top and middle, with a larger columnar elongated grain at the bottom near the substrate.
3. The coating hardness values exhibited approximately threefold greater values compared to the substrate hardness values across all cladding conditions. Microhardness values of 540 ± 10 HV_0.1_ were obtained at an ultrasonic amplitude of 5 µm, which was higher than the values of 505 ± 10 HV_0.1_, 490 ± 10 HV_0.1_, and 460 ± 10 HV_0.1_ recorded with amplitudes of 15 µm, WOU, and WU of 10 µm, respectively. The increased microhardness at 5 µm is attributed to ultrasonic vibrations’ acoustic and cavitation effects.
4. The friction coefficient was found to be stable and at its lowest when using an ultrasonic amplitude of 5 µm, compared to greater amplitudes of WOU and WU. The 3D surface profile of the wear reveals that ultrasonic assistance at a 5 µm amplitude results in a lower scar depth. This indicates that the coating generated with the assistance of ultrasonic vibrations has excellent antifriction capabilities, and the wear mechanism was dominated by abrasive and oxidative wear with fewer grooves and debris.

## Figures and Tables

**Table 1:** AlCrFeMnNi chemical composition of principle alloying elements.

Elements	Al	Cr	Fe	Mn	Ni
At%	20.90	19.24	18.03	22.25	19.58
Wt%	11.41	20.24	20.36	24.73	23.26

**Table 2:** Properties of elements in the high-entropy alloy of AlCrFeMnNi.

Elements	Al	Cr	Fe	Mn	Ni
Melting point (°C)	660	1857	1535	1277	1453
Vaporization heat (kJ/Mol)	238.8	339.8	349.9	220.7	374.5
Atomic radii (nm)	1.432	1.27	1.241	1.32	1.24

**Table 3:** EDS analysis of coating at different points on the coating surface.

Elements (at. %)	Fe	Al	Ni	Mn	Cr	C
Point 1	40.40	6.66	10.40	3.96	15.77	22.79
Point 2	45.42	2.35	7.46	2.31	14.91	27.56
Point 3	37.38	8.48	14.44	5.76	14.61	19.32
Point 4	47.33	1.96	6.34	2.34	17.41	24.63
Point 5	42.15	5.46	13.91	7.24	15.11	16.14

**Table 4:** EDS analysis of wear tracks at different points on the wear surfaces.

Elements (at. %)	O	Fe	Al	Ni	Mn	Cr	C
Point 1	12.83	24.98	4.17	14.49	7.30	25.72	10.51
Point 2	30.52	28.23	9.81	7.46	2.44	9.04	12.50
Point 3	4.84	39.34	6.81	9.76	6.35	17.11	15.79
Point 4	24.24	29.50	6.84	6.03	5.95	11.88	15.60

## Data Availability

Data are contained within the article.
